# Bipolar Switching Properties of Neodymium Oxide RRAM Devices Using by a Low Temperature Improvement Method

**DOI:** 10.3390/ma10121415

**Published:** 2017-12-12

**Authors:** Kai-Huang Chen, Ming-Cheng Kao, Shou-Jen Huang, Jian-Zhi Li

**Affiliations:** 1 Department of Electrical Engineering and Computer Science, Tung Fang Design University, Kaohsiung 82941, Taiwan; patrick@mail.tf.edu.tw; 2 Department of Electronic Engineering, Hsiuping University of Science and Technology, Taichung 41280, Taiwan; kmc@mail.hust.edu.tw; 3 Department of Electronic Engineering, Southern Taiwan University of Science and Technology, Tainan 71005, Taiwan; d9131802@mail.tf.edu.tw

**Keywords:** SCF, bipolar switching properties, neodymium oxide, thin film, RRAM

## Abstract

Bipolar resistive switching properties and endurance switching behavior of the neodymium oxide (Nd_2_O_3_) thin films resistive random access memory (RRAM) devices for a high resistive status/low resistive status (HRS/LRS) using a low temperature supercritical carbon dioxide fluid (SCF) improvement post-treatment process were investigated. Electrical and physical properties improvement of Nd_2_O_3_ thin films were measured by X-ray diffraction (XRD), scanning electron microscopy (SEM), X-ray photoelectron spectroscopy (XPS), and current versus voltage (*I-V*) measurement. The metal-like behavior of ohmic conduction mechanism and metallic cluster reaction of hopping conduction mechanism in initial metallic filament path forming process of the SCF-treated thin films RRAM devices was assumed and discussed. Finally, the electrical conduction mechanism of the thin films RRAM derives for set/reset was also discussed and verified in filament path physical model.

## 1. Introduction

Many post-treatment fabrication processes for re-crystalline structure and polycrystalline quantities properties improvement of material structure of various oxide thin films were widely consideration and investigated. In past, the rapid thermal annealing (RTA), the conventional furnace annealing (CFA), and low temperature polycrystalline (LTPS) process was an important indispensable and essential technology for physical and electrical properties improvement. However, the high fabrication temperature, long process time, high pollution reaction, and thermal budget effect were serious consideration problems for integrated circuits (IC) process. To traps and oxygen vacancy efficiently decreased and passivated, no damaging diffusion effect into thin film, the excellent properties of liquid-like supercritical carbon dioxide fluid treatment (SCF) method on physical and electrical properties of thin films resistive random access memory (RRAM) devices were widely attracted considerable method [1,2,3,4,5].

The static random access memory (SRAM), dynamic random access memory (DRAM), ferroelectric random access memory (FeRAM), magnetron memory (MRAM), and phrase change memory (PCM) devices were studied and were used for applications in various portable electrical devices [1,2,3,4,5,6,7,8,9]. However, the different oxide thin film RRAM devices were widely investigated and discussed because of its non-volatility, long retention cycles, high storage capacity, low power consumption, and high speed readout characteristics [10,11,12,13,14]. In addition, excellent and stable endurance and retention properties of the suitable thin film RRAM devices were widely continued considerable and investigated. However, rare earth thin films for neodymium oxide was not yet discussed and found. 

To further discuss the bipolar resistive switching properties and initial metal metallic filament forming mechanism of neodymium oxide thin films, the Al/Nd_2_O_3_/ITO structure device was fabricated for indium tin oxide (ITO) and aluminum (Al) electrode. In addition, the ohmic conduction, schottky emission condition, and hopping conduction of thin film RRAM devices for low resistive state (LRS) and high resistive state (HRS) was discussed by *I-V* curves fitting. 

## 2. Material and Methods

To overcome the existence of the oil pollution and partial problem, the defect and metal ion on ITO substrate were removed during standing clean process. To remove the defects of metal target and obtain stable plasma during deposition time, pre-sputtering time of non-treated thin films was maintained for 30 min under argon gas ambient. The optimal sputtering parameters, such as, the sputtering power of 160 W, different oxygen concentration parameters, and chamber pressure of 10mTorr. After thin films depositing process, the neodymium oxide thin film was post-treated by super supercritical CO_2_ fluid process (SCF). In addition, the neodymium oxide thin films were placed in a supercritical fluid system at 150 °C for 2 h, it was injected with 3000 psi SCF fluids that were mixed with 5 vol % pure H_2_O and 5 vol % propyl alcohol. Each of the experimental parameters detail design and selection were considered and determined by grey entropy strategy of situation analysis method. To complete the RRAM MIM (Metal-Insulator-metal, MIM) structure, arrays of circular top contacts are formed by depositing Al film of 500 nm by dc sputtering technology. The typical *I-V* switching curves of RRAM devices were obtained by Agilent B1500 semiconductor parameter analyzer. To investigate the super supercritical CO_2_ fluid treatment process in neodymium oxide thin films, the physical properties was proved and analyzed by the SEM, XPS, and XRD measurement.

## 3. Results and Discussion

**Figure 1:**
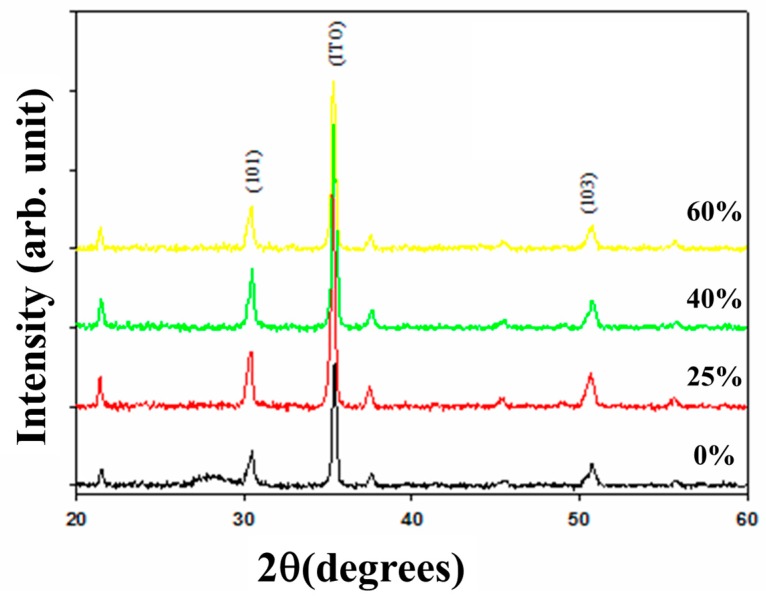
The X-ray diffraction (XRD) patterns of the neodymium oxide thin films for the different oxygen concentration parameters.

**Figure 2:**
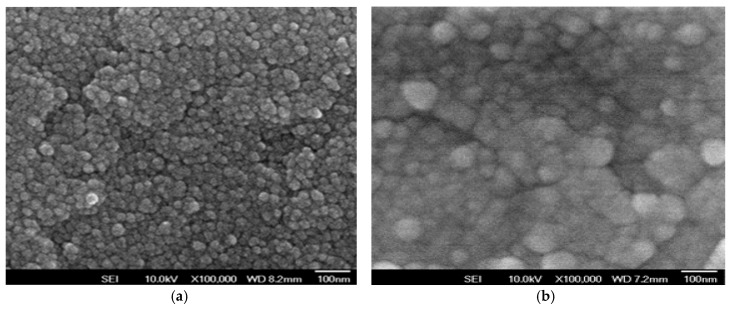
The SEM morphology of the neodymium oxide thin films for (**a**) 40%, and (**b**) 60% oxygen concentration parameters.

In XRD patterns, the (101) and (103) preferred peaks of neodymium oxide thin films was observed and discussed in Figure 1. All of the thin films were exhibited the polycrystalline structure for the different oxygen concentration parameters. In addition, the (101) preferred peaks and a smallest full width at half maximum (FWHM) value of thin films for 40% oxygen concentration were preferred. In addition, the crystallinity and grain size of thin films was measured and analyzed from a smallest FWHM value to others. Besides, the micro-structure and grain size of neodymium oxide thin films for 40% and 60% oxygen concentration parameters all exhibited the round and circle style in Figure 2. The grain size of thin film for 60% oxygen concentration was examined about 80 nm. In previous study, the grain growth, the dielectric loss, and leakage current properties of non-treated thin films were exhibited apparent improvement in different oxygen annealing treatment process. The trap and vacancy decreased of leakage current density lowing was efficiently improvement by the oxygen annealing process.

**Figure 3:**
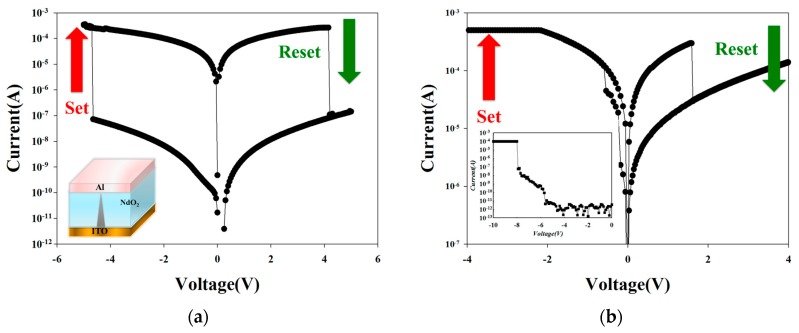
Bipolar switching curves of the neodymium oxide thin films for (**a**) 40%, and (**b**) 60% oxygen concentration parameters.

To define the set/reset process, the neodymium oxide RRAM device transferred to LRS for applied a high positive bias than the set voltage was called the set process. In addition, the operation current continuously decrease from LRS to HRS for the applied negative bias over the reset voltage was called the reset process. Figure 3a,b presented the 100 cycles *I-V* switching curves of the neodymium oxide thin films RRAM device for 40% and 60% oxygen concentration parameters. For 40% oxygen concentration parameters, the *I-V* curves were exhibited the symmetry and equal bipolar switching properties. The maximum memory ratio was about 10^3^ ratio. This effect caused by excess oxygen vacancy and un-bonded defect was appropriately recombination for 40% oxygen concentration environment. However, asymmetry and inverted *I-V* switching curves of 60% oxygen concentration neodymium oxide thin films RRAM device was discussed. The inverted *I-V* curves behavior was might cause by excess oxygen ions early into ITO bottom electrode during thin film deposition process [13,14,15,16].

**Figure 4:**
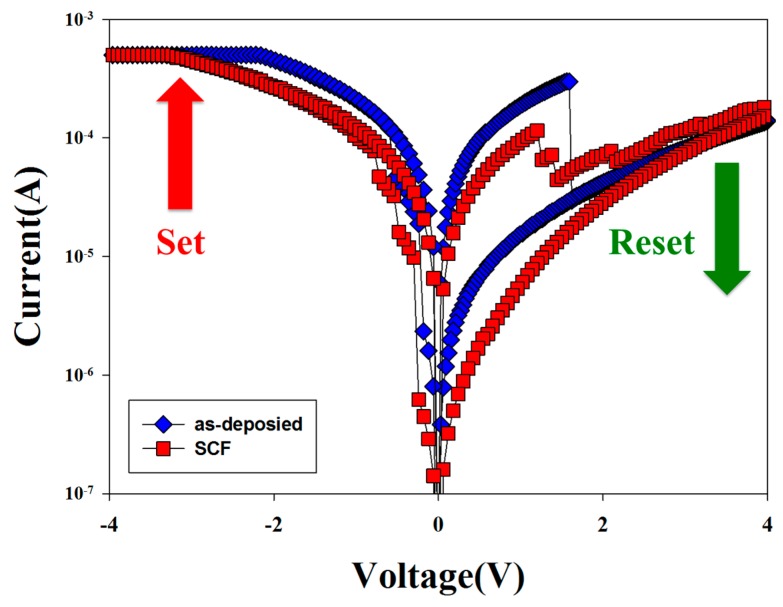
Bipolar switching curves of non-treated and supercritical carbon dioxide fluid (SCF)-treated neodymium oxide thin films.

Figure 4 shows the 100 cycles *I-V* switching curves of the non-treated and SCF-treated neodymium oxide thin films RRAM device. For HRS/LRS, the operation current of the SCF-treated thin film RRAM devices was apparently decreased because of its trap, defects, and oxygen vacancy decrease. The on/off ratio operation current of SCF-treated neodymium oxide thin films RRAM in LRS were slightly apparently increased and the oxygen vacancy efficiency improvement by SCF post-treatment process. The electrical conduction mechanism of the non-treated and SCF-treated thin films RRAM devices was proved in *I-V* curves fitting later.

**Figure 5:**
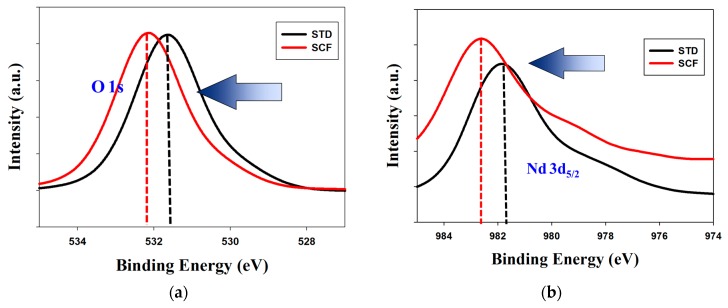
The X-ray photoelectron spectroscopy (XPS) curves of the (**a**) O 1s, and (**b**) Nd 3d_5/2_ for SCF-treated and non-treated neodymium oxide thin films.

For XPS results that were obtained, the oxygen (O 1s) and neodymium (Nd 3d_5/2_) dangling bonds of SCF-treated neodymium oxide thin films was slightly shifted to high banging energy in Figure 5a,b. As shown in Figure 5, we suggested that the O–H dangling bonds in non-treated thin film was efficiently decreased and repaired the traps and defects using a supercritical carbon dioxide fluid treatment method. The on current of the low resistive state (LRS) of the SCF treated neodymium oxide thin film was decreased and was attributed to the Nd–O–Nd bonds increased and neodymium to oxidize.

To ohmic conduction equation,

(1) $$J=N_{o}e\mu \frac{V}{d}$$

and space charge limited conduction (SCLC) conduction equation,

(2) $$J=\frac{9}{8}\varepsilon \mu \frac{V^{2}}{d_{3}},$$

where *J* is the current density, *N_o_* is thermal carrier concentration, *ε* is the insulator permittivity, *V* is applied voltage, and *d* is films thickness. In addition, the schottky emission equation,

(3) $$J=A^{*}T^{2}\exp[-q(\Phi_{B}-\sqrt{\frac{qE_{i}}{4\pi \varepsilon_{i}}})/kT],$$

where *T* is the absolute temperature, Φ*_B_* is the schottky barrier height, *ε_i_* is the insulator permittivity, *K* is Boltzmann’s constant, and A* is Richardson constant. The hopping conduction,

(4) $$J=qN_{a}v_{o}e^{-q\Phi_{T}/kT}e^{qaV/2dkT},$$

where *N_a_*, Φ*_T_*, *v_o_*, and *d* are density of space charge, mean of hopping distance, barrier height of hopping, intrinsic vibration frequency, and film thickness, respectively, To further prove the electrical conduction mechanism in metallic filament process of RRAM devices, the ohmic conduction, schottky emission conduction, and hopping conduction were be defined by *lnI-lnV* and *lnI-V*^1/2^, and *lnI-V* curves fitting [17].

**Figure 6:**
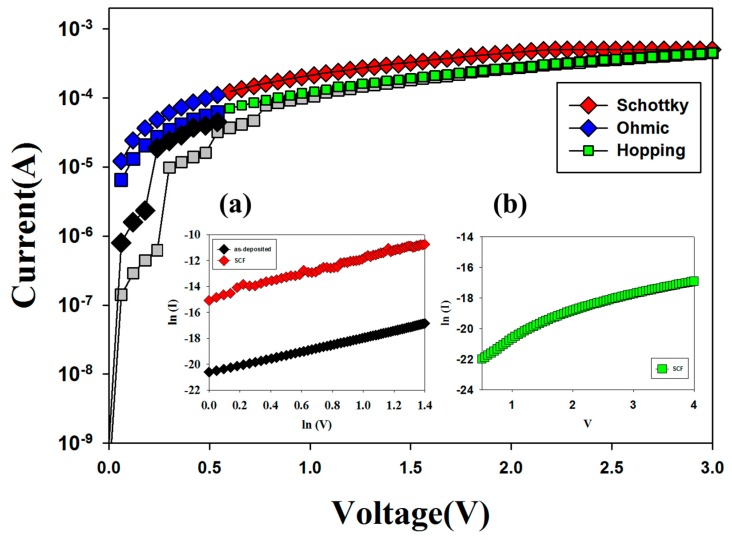
The electrical conduction mechanism of the SCF-treated and non-treated neodymium oxide thin films for (**a**) ohmic conduction and (**b**) hopping conduction mechanism.

**Figure 7:**
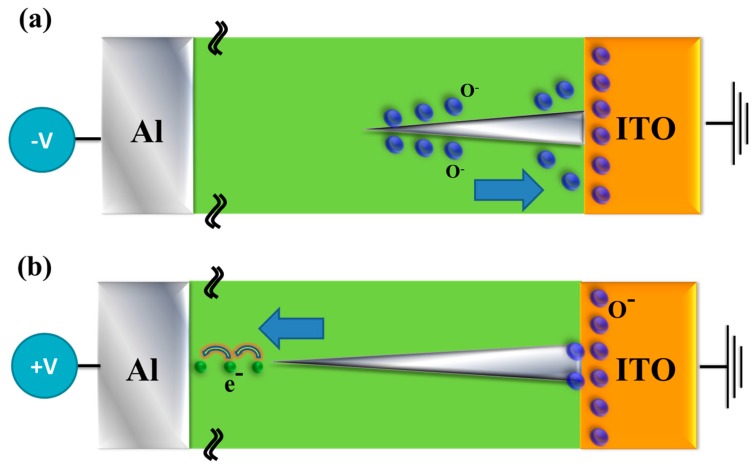
Electrical transferred mechanisms and initial metallic filament path diagram of the thin films resistive random access memory (RRAM) devices for (**a**) reset, and (**b**) set process.

Figure 6 shows the original *I-V* curves of neodymium oxide thin films RRAM devices in HRS for black and grey box. For LRS in Figure 6a,b by *lnI-lnV* and *lnI-V* curves fitting, the *I-V* curves of neodymium oxide thin films RRAM devices for 40% and 60% oxygen concentration parameters exhibited the ohmic conduction in low applied voltage. In addition, the shallow trapped electrons jumped to the energy active barrier and forming leakage current in metallic filament conduction path of SCF-treated RRAM devices was exhibited the hopping conduction mechanism by *lnI-V* curves fitting in Figure 6b. In initial metallic filament forming model, the metal element separated and clustered in metallic filament conduction paths of neodymium oxide thin films was assumed and explained in Figure 7. In Figure 7, the grey park was assumed to metallic filament path for different applied voltage direction. Wavy lines were actual thickness thin film.

**Figure 8:**
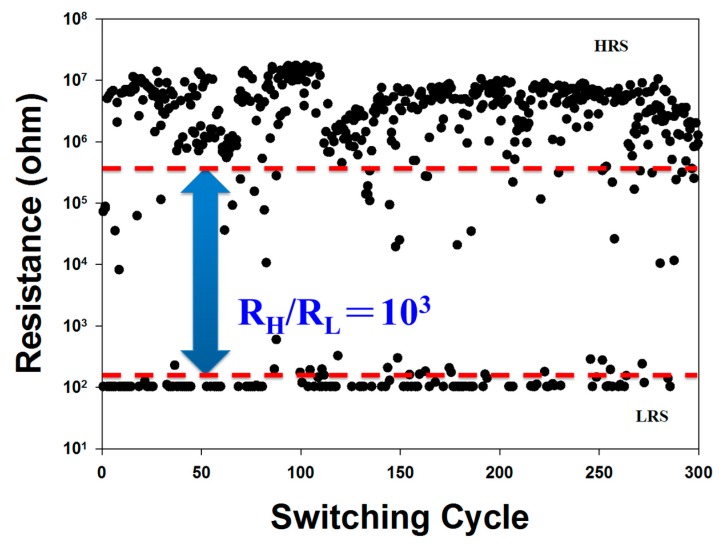
Endurance characteristics of the SCF-treated thin films RRAM devices at room temperature as measured at 0.5 V.

To define the excellent nonvolatile random memory devices, the on/off ratio retention characteristics, the switching cycling testing of reliability and retention characteristics measurement for SCF post-treatment RRAM devices for HRS/LRS were presents and measured in Figure 8. 

In Figure 8, the black circle and redline was about resistance value for HRS/LRS. In addition, the significant and apparently stable properties for on/off switching cycling testing in SCF-treated neodymium oxide thin film RRAM devices for HRS/LRS were observed for the further non-volatile characteristics calculation. 

## 4. Conclusions

The low temperature, liquid-like, and excellent properties of the supercritical carbon dioxide fluid technology was an efficiently improvement method for its low thermal budget, simply post-treatment, and re-oxidation ability properties. In initial metallic filament forming process, the bipolar resistive switching behavior and electrical conduction mechanism of SCF post-treatment RRAM devices for HRS/LRS state were discussed and fitting by the electron conduction path model.

The defects and oxygen vacancy of non-treated neodymium oxide thin films for different oxygen concentration parameters were also effectively achieved and improved by the of supercritical carbon dioxide fluid treatment. The non-treated and SCF-treated neodymium oxide thin films RRAM devices for LRS were exhibited the ohmic conduction, schottky conduction emission, and hopping conduction model in low and high applied voltage. Finally, the inverted and symmetry bipolar *I-V* switching curves of SCF-treated neodymium oxide thin films RRAM devices were apparently determined and observed because of the different oxygen ions quantity exist in initial metallic forming paths behavior.
